# Impacts of a Produce Prescription Program on Food Security, Diet Quality, and Psychosocial Health of Adults with Medicaid and Chronic Health Conditions: A 12-Month Longitudinal Evaluation

**DOI:** 10.1007/s11606-026-10390-x

**Published:** 2026-04-14

**Authors:** Rachel Zimmer, Lindsey Abdelfattah, Diane Shenberger, Charlotte Crotts, Sarah Birken, Amresh Hanchate

**Affiliations:** 1 Department of Implementation Science, Division of Public Health Sciences, Wake Forest University School of Medicine, Winston Salem, NC USA; 2 Section of Gerontology and Geriatric Medicine, Department of Internal Medicine, Wake Forest University School of Medicine, Winston-Salem, NC USA; 3 Department of Social Sciences and Health Policy, Division of Public Health Sciences, Wake Forest University School of Medicine, Winston-Salem, NC USA; 4 YMCA of Northwest North Carolina, Winston Salem, NC 27101 USA

**Keywords:** food insecurity, produce prescription, food is medicine, medicaid, behavioral health, nutrition, hypertension, implementation science

## Abstract

**Background:**

Food insecurity (FI) is a driver of chronic disease. Produce prescription (PRx) interventions pair access to produce with education, but evidence among Medicaid-insured adults remains limited.

**Objective:**

Evaluate the impact of PRx on FI, fruit and vegetable (FV) intake, and psychosocial and clinical outcomes among adults with chronic conditions.

**Design:**

Prospective, non-randomized cohort study with a matched comparison group.

**Participants:**

A total of 125 Medicaid-insured, food-insecure adults with chronic metabolic conditions were enrolled in the intervention cohort and 92 matched to the Supplemental Nutrition Assistance Program (SNAP)-only cohort.

**Intervention:**

Weekly home-delivered produce, registered dietitian-led coaching, and education over 12 months.

**Main Measures:**

FI (USDA 10-item Food Security Survey Module), FV intake (National Cancer Institute screener), depression (PHQ-9), loneliness (UCLA 3-item), fatigue (FACIT-F), healthcare utilization and costs, and blood pressure (BP). For the comparison cohort, some measures were obtained for 6 months of follow-up.

**Key Results:**

At 6 months, based on between-group differences, PRx was associated with greater improvements in FV intake (difference in change: + 0.3 vs. + 0.03 cups/day,* p* = 0.002) and greater reductions in loneliness (difference in change: −0.6 points, *p* = 0.022). Between-group differences for FI (−8.2 percentage points, *p* = 0.26) and moderate-to-severe depression (−10.8 percentage points, *p* = 0.07) favored PRx but were not statistically significant. At 12 months, no significant between-group differences in healthcare utilization and costs were observed. Among the 105 (84%) intervention participants who completed 12-month follow-up, between the pre- and post-intervention periods, FI declined by 17.1% (*p* = 0.003), FV intake increased by 0.4 cups/day (*p* < 0.001), fatigue improved by 3.1 points (*p* = 0.019), and among participants with elevated baseline BP (*n* = 73), systolic BP decreased by 8.6 mmHg (*p* = 0.003).

**Discussion:**

In this study, PRx was associated with improved diet quality and loneliness compared with a SNAP-only comparison. Within-group analyses demonstrated improvements across dietary, psychosocial, and BP outcomes, supporting further evaluation using randomized or comparative designs.

## INTRODUCTION

Food insecurity (FI), limited or uncertain access to adequate nutrition, is a growing public health concern in the USA. In 2024, an estimated 13.7% of the population (over 47 million people) experienced FI, up from 12.8% in 2022, reflecting the expiration of pandemic-era relief, rising food prices, and persistent structural inequalities.^1,2^ FI is associated with poor dietary quality, greater risk of diabetes and hypertension, and higher healthcare utilization and costs.^3–6^

FI also has implications for mental health. Food-insecure adults have a higher prevalence of depressive symptoms, anxiety, social isolation, and stress, with dose–response gradients whereby greater FI severity is linked with worse mental health.^7–9^ Among people with chronic conditions, FI is associated with lower self-efficacy, poorer adherence, poorer control of chronic disease, and higher costs.^10,11^ US families experiencing FI incur approximately 20% greater total healthcare expenditures than food-secure families, an adjusted annual difference of about $2456 per family.^10^

FI is increasing among older adults and remains highly prevalent among Medicaid beneficiaries. In 2023, 9.3% of US households with an adult aged ≥ 65 reported FI, up from 7.1% in 2021.^7,12–15^ More than one-third of Medicaid beneficiaries report reducing food quality or quantity due to cost.^16,17^ Among both populations, FI is associated with poorer chronic disease control, higher rates of depression, and increased healthcare utilization and costs.^18^

“Food is Medicine” (FIM) interventions, which integrate healthy food access and nutrition education into healthcare, have emerged as effective strategies to address dietary drivers of chronic disease.^19,20^ Within this movement, produce prescription programs (PRx) provide targeted support by offering produce vouchers or direct provision, typically prescribed by clinicians, and coupled with nutrition education or coaching.^21–23^ Programs typically allocate $10 to $50 per week, with redemption sites ranging from farmers’ markets to grocery stores.^21–25^

Evidence increasingly supports PRx for improving diet and FI among low-income adults with chronic conditions.^23–27^ A recent review found that > 70% of PRx studies increased fruit and vegetable (FV) intake (mean + 0.85 cups/day), and reduced FI odds by about one-third.^25^ Some studies also report cardiometabolic benefits (e.g., reductions in blood pressure and A1c), though results are mixed, reflecting differences in populations, dose, and intensity.^23,27^ Mental health impacts remain underexplored, and few evaluations focus on Medicaid beneficiaries.^17,28,29^ There is a bidirectional relationship between FI and mental health: FI can worsen anxiety and depression, while poor mental health can hinder self-management, making assessment of psychosocial outcomes critical.^6,11,22^

This study addresses these gaps by evaluating Fresh Food Rx (FFRx), a community-health system partnership designed to address dietary, psychological, and cardiometabolic risk factors among Medicaid-insured adults experiencing FI. Grounded in behavioral theory, FFRx integrates produce access with individualized and group-based nutrition support.^33,34^ Using a prospective, matched cohort design, this study evaluated the impact of FFRx on food security, diet quality, psychological outcomes, healthcare utilization, and cardiovascular risk over 12 months. 

## METHODS

### Study Design

We conducted a mixed-methods, non-randomized, prospective longitudinal cohort study with a matched comparison group between February 2023 and June 2024 in Forsyth County, NC, and approved by the Institutional Review Board of the Wake Forest School of Medicine (IRB00089384).

### Intervention: Fresh Food Rx

Fresh Food Rx was a 12-month, multi-component PRx intervention informed by the Capability, Opportunity, Motivation (COM-B) model and the Theoretical Domains Framework (TDF)^32–34^ (see Table 1). Participants received weekly home-delivered produce boxes valued at $25 per week and selected from standard, mixed, or culturally tailored box options. In addition, participants were offered up to three individualized nutrition coaching sessions with a registered dietitian (RD) using motivational interviewing and study-developed curricula and were invited to quarterly group educational sessions addressing topics such as budget-conscious shopping, cooking, seasonal produce preparation, and meal planning. Ongoing engagement was supported through newsletters and brief reminders. At study completion, participants were provided a list of food resources and produce vouchers valued at $50.

**Table 1:** Fresh Food Rx Core Functions, Hypothesized Mechanisms of Action, and Implementation

Core function	Inputs	Core activities	Outcomes/measures	Motivators (COM-B/TDF)^34,35^	Data source and timing	Implementation notes
Referrals into program	Study flyers, RD at YMCA, primary care providers	Identification of eligible adults with Medicaid, FI (2-item Hunger Vital Sign), and at least one diet-related chronic condition; referrals from self-referral from flyers, YMCA RD referrals, or healthcare provider referral	Number and source of referrals; number eligible; number enrolled	**Opportunity:** Expands social and system opportunity to engage high-risk patients **TDF:** Environmental context and resources; social influences	Referral logs (ongoing); screening logs prior to baseline	Outreach to high ADI neighborhoods and safety-net clinics; simple referral workflow for providers
Access to fresh produce	Grant funding to support produce costs; Produce Box Co.; logistics partners; delivery staff	Weekly home‑delivered produce boxes ($25 value) with participant choice of a box each week with: (1) 6–7 different produce items; (2) 4–5 produce items + eggs; or (3) 6–7 produce items tailored for Latine culture	FI (USDA 10-item FSSM)^26^; FV intake (NCI FV screener)^27^ # boxes delivered; % weeks received; missed deliveries; systolic and diastolic blood pressure; weight; healthcare utilization	**Capability/opportunity:** Reduces financial and geographic barriers; increases physical opportunity to obtain and consume FV **TDF:** Environmental context and resources	Program delivery logs weekly; participant surveys baseline, 6 and 12 months	Prioritize high‑ADI areas; accommodate delivery windows; incorporate participant feedback loops on produce type, quality, and cultural relevance
Nutrition knowledge and skills	RD with motivational interviewing training; study developed, individually tailored curricula	Up to 3 individualized RD coaching sessions focused on goal‑setting, motivational interviewing, and diet education using study-developed, evidence-based nutrition materials	FV intake (NCI FV Screener)^27^; FI (USDA 10-item FSSM)^26^; PHQ‑9 (depressive symptoms)^28^; UCLA‑3 (loneliness)^29^; FACIT‑F (fatigue)^32^ % sessions complete; systolic and diastolic blood pressure; weight; healthcare utilization	**Capability/motivation:** Builds psychological capability (knowledge, skills) and reflective motivation (goals, beliefs about capabilities) **TDF:** Knowledge; skills; beliefs about capabilities**;** goals	RD notes; attendance logs; participant surveys at baseline, 6 and 12 months	Offer in person, phone, video; literacy‑appropriate materials; interpreter access; individualized goal setting
Educational Sessions	Community-partners; RD; community venues, virtual platform	Quarterly group educational sessions (e.g., budget-conscious shopping, cooking demonstration, seasonal produce preparation, gardening, physical activity, meal planning)	PHQ-9^28^ UCLA-3^29^ session satisfaction surveys; % education sessions attended	**Social opportunity and capability:** Builds cooking skills and confidence; fosters peer learning and social support for healthy eating **TDF:** Skills; social influences; social/professional role and identity	Attendance log; satisfaction survey baseline, 6 and 12 months; qualitative interviews	Scheduled at accessible times; culturally relevant education; community-located with transportation support; virtual option
Cues and reinforcement	Monthly newsletters; RD-curated seasonal recipes; reminders	Monthly newsletters packaged with boxes; brief text/phone reminders from study staff and Produce Box Co. to reinforce goals, promote session attendance, remind of delivery time, and prompt use of produce and recipes	Newsletter reach; satisfaction surveys; self-reported use of recipes and tips	**Automatic motivation:** Repeated cues and prompts reinforce habit formation and ongoing engagement **TDF:** Reinforcement, memory, attention, and decision processes	Distribution logs monthly; surveys every 3 months	Visually engaging, plain language design; aligned with seasonal produce box contents; brief, actionable tips

*FI*, Food insecurity (USDA FSSM 10-item)^38^; *FV*, fruit and vegetable intake, cups/day (NCI FV screener)^39^; *PHQ9*, Patient Health Questionnaire-8 for depressive symptoms^40^; *UCLA3*, 3-item UCLA Loneliness Scale^41^; *FACITF*, Functional Assessment of Chronic Illness Therapy-Fatigue^42^; *RD*, registered dietitian; *ADI*, area deprivation index; *COM-B*, Capability, Opportunity, Motivation, Behavior; *TDF*, Theoretical Domains Framework^34,35^

### Intervention Cohort

**Figure 1:**
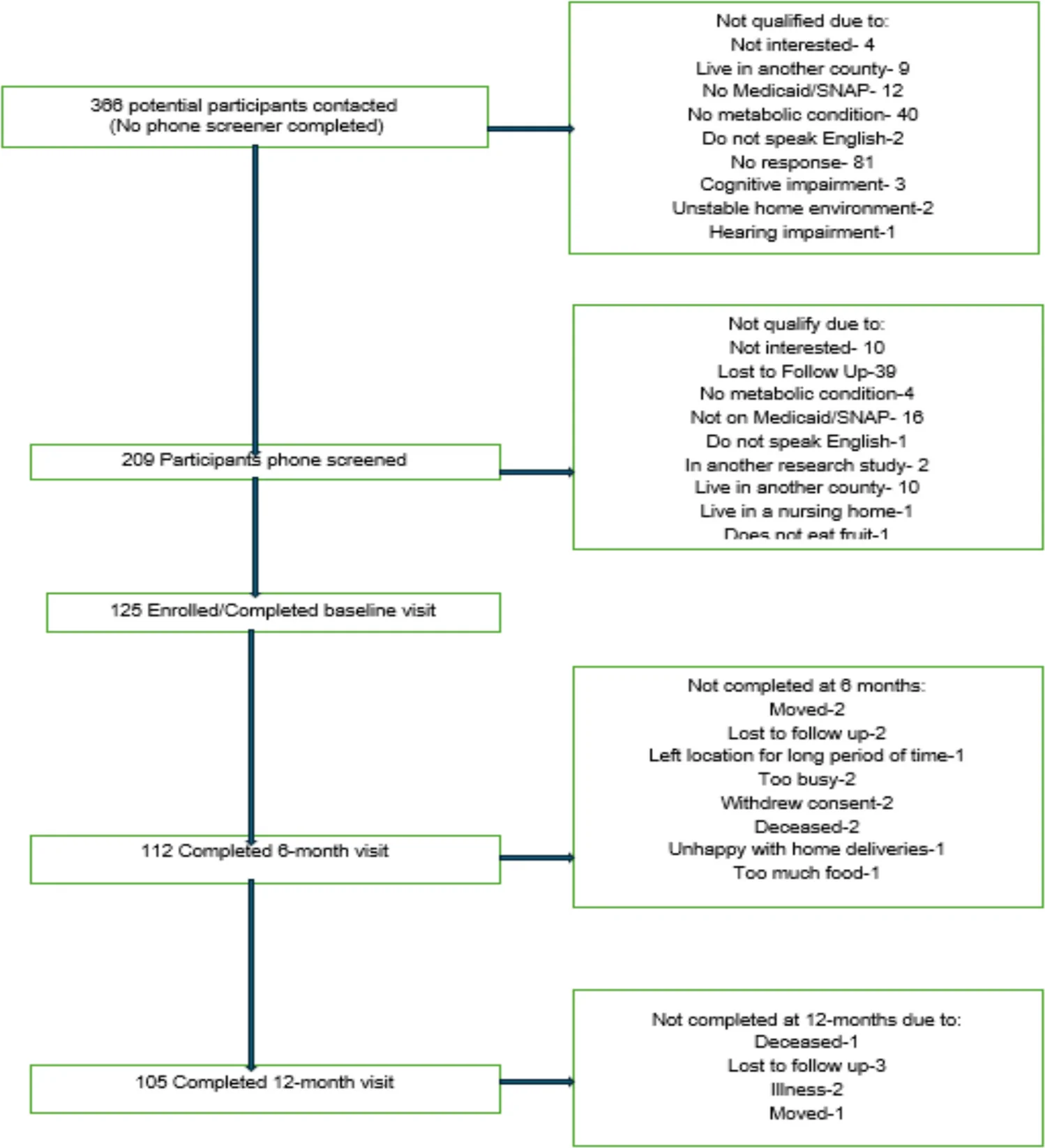
Fresh Food Rx recruitment map.

We enrolled 125 English-speaking adults with Medicaid, FI (per 2-Question Hunger Vital Sign),^35^ and at least one diet-related chronic condition (e.g., diabetes, hypertension) (see Fig. 1). Participants were identified via self-referral from flyers, referral from registered dietitian (RD) at YMCA, or referral from clinicians when patients screened positive for FI and had a diet-related health condition (e.g., diabetes, hypertension, pre-diabetes, obesity).^32^

### Comparison Cohort

**Figure 2:**
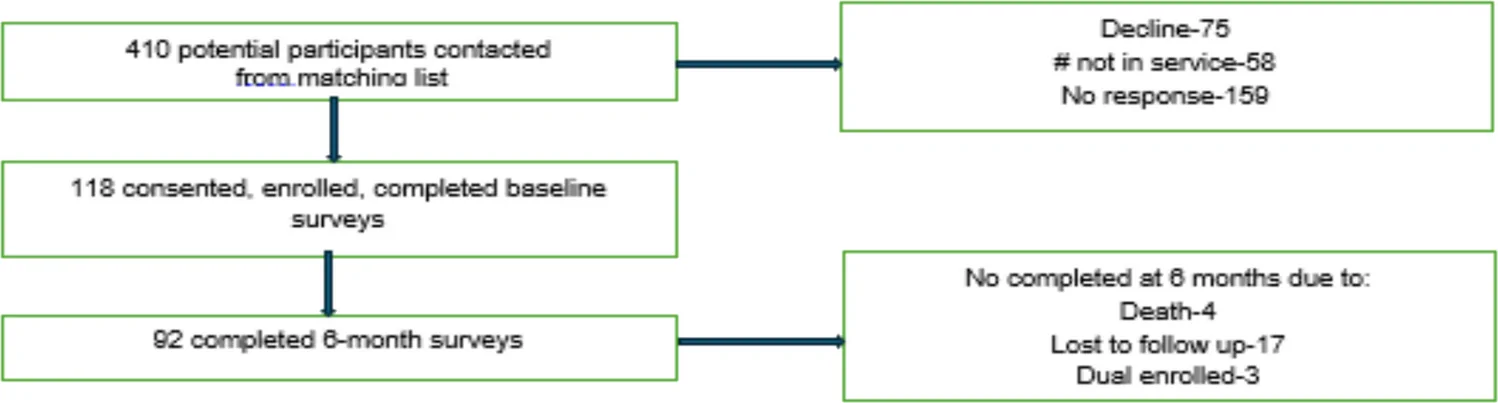
SNAP participant recruitment map.

A matched group of 92 SNAP recipients was identified from the same health system’s electronic health records database. Using the propensity score-based matching approach described by Rigdon et al., we matched intervention participants to SNAP-only recipients residing in Forsyth County who received care within the same health institution prioritizing balance on age, sex, race, ethnicity, neighborhood percent living in poverty, insurance, and presence of similar health conditions^36^ (see Fig. 2). Participants received no nutrition intervention but were given local food resources after completion of the baseline survey. A SNAP-only comparison group was selected to contextualize secular and policy-related changes occurring alongside ongoing nutrition assistance.

### Data Collection

Intervention participants completed in person study visits at baseline, and at 6 and 12 months, during which trained research staff administered surveys and obtained clinical assessments using standardized protocols. Comparison cohort participants completed surveys virtually at baseline and 6 months. Primary outcomes assessed in both cohorts included (1) FI was measured using the USDA 10-item Adult Food Security Survey Module (FSSM) which categorizes households as food secure, marginally food secure, or food insecure (low or very low food security).^37^ (2) Dietary intake was assessed using the National Cancer Institute (NCI) Fruit and Vegetable Screener, scored to estimate total fruit and vegetable intake in cups per day, with higher scores indicating greater intake.^38^ (3) Depressive symptoms were measured using the Patient Health Questionnaire-9 (PHQ-9), a validated instrument assessing depressive symptom severity; scores ≥ 10 indicate moderate depression and ≥ 15 severe depression.^39^ (4) Loneliness using the UCLA Loneliness Scale (three-item short-form), assessed feelings of loneliness, with higher scores indicating greater loneliness.^40^ (5) Healthcare utilization was obtained from electronic health records and included number of primary care visits, hospital admissions, and emergency room use visits; corresponding costs were estimated using national average Medicare reimbursement rates.^41,42^

Secondary outcomes, assessed in the intervention cohort only at baseline, and at 6 and 12 months, included fatigue using the Functional Assessment of Chronic Illness Therapy-Fatigue (FACIT-F) with higher scores indicating lower fatigue, and clinical measures (weight, waist circumference, and blood pressure) collected using standardized protocols.^43,44^

Programmatic data included participant attendance and satisfaction with intervention components. Demographics, household composition, and socioeconomic data were collected at baseline. Retention was defined as completion of the 12-month follow-up assessment among participants with baseline data.

### Statistical Analysis

The study sample size was determined a priori based on feasibility and available enrollment and was sufficient to detect small-to-moderate between-group differences in primary outcomes at 6 months. With 125 intervention participants and 92 matched comparison participants, the study had approximately 80% power to detect small-to-moderate standardized differences (Cohen’s *d* ≈ 0.40–0.45) between groups for continuous outcomes, assuming a two-sided *α* of 0.05. Power to detect differences for binary outcomes was more limited. Within-intervention cohort analyses were conducted to describe longitudinal changes over time among participants who completed follow-up assessments. These analyses were not powered to detect causal effects. Analyses restricted to participants with elevated BP were conducted as exploratory subgroup analyses and were not powered for formal between-group comparisons.

We compared the demographic and comorbidity characteristics (at baseline) of the intervention and the comparison cohorts. Linear regression models with individual fixed effects were used to estimate within-individual changes in outcomes over time. For the primary outcomes measured for both the intervention and comparison cohorts, we compared the between-group pre- to post-intervention change in outcomes for the two cohorts. To estimate the change in outcomes associated with the intervention, we included a dichotomous indicator of the post-intervention period (0/1) and the interaction of the post-intervention indicator with the indicator of individuals in the intervention cohort (0/1). The coefficient of the interaction term is identified as the change in outcome associated with the intervention. We estimated separate regression models for distinct post-intervention periods (6-month, 12-month, and 18-month). For secondary outcomes that were only obtained for the intervention cohort, we estimated the average within-group change using a linear regression model with individual fixed effects and a covariate indicator of post-intervention period. We obtained standard errors robust to heteroskedasticity. All analyses were conducted in Stata/MP v17 with a significance threshold of *p* < 0.05.

## RESULTS

Results are presented first for prespecified between-group comparisons at 6 months, followed by descriptive within-intervention group cohort analyses and exploratory subsample analyses.

### Participant Characteristics and Program Engagement

A total of 125 Medicaid-insured adults with FI and at least one diet-sensitive chronic condition were enrolled in the intervention cohort, and 92 matched SNAP-only participants comprised the comparison cohort. At baseline, intervention participants were predominantly female (74.1%) and Black (59.8%), with a mean age of 56.8 years. Over half (52.7%) had a diagnosis of diabetes; 67% had HTN. Nearly 30% screened positive for moderate-to-severe depression, and approximately 91% resided in neighborhoods with high Area Deprivation (ADI 76 to 100). Baseline demographic and clinical characteristics were generally comparable between cohorts following propensity score matching^45^ (see Table 2).

**Table 2:** Baseline Characteristics in Intervention and Control Group

Characteristics	Fresh Food Rx	SNAP	Total	*p*-value
*N*	112 (54.9%)	92 (45.1%)	204 (100.0%)	
Self-identified sex				
Male	28 (25.0%)	16 (17.4%)	44 (21.6%)	0.420
Female	83 (74.1%)	75 (81.5%)	158 (77.5%)	
Unknown/missing	1 (0.9%)	1 (1.1%)	2 (1.0%)	
Age, mean (standard deviation)	56.812 (12.563)	55.717 (11.656)	56.319 (12.145)	0.523
Age group (*N* = 5)				
18–34	6 (5.4%)	4 (4.3%)	10 (4.9%)	0.563
35–44	14 (12.5%)	13 (14.1%)	27 (13.2%)	
45–54	19 (17.0%)	19 (20.7%)	38 (18.6%)	
55–64	40 (35.7%)	38 (41.3%)	78 (38.2%)	
65 +	33 (29.5%)	18 (19.6%)	51 (25.0%)	
Race/ethnicity (*N *= 4)				
Non-Hispanic White	25 (22.3%)	29 (31.5%)	54 (26.5%)	0.172
Non-Hispanic Black	67 (59.8%)	55 (59.8%)	122 (59.8%)	
Hispanic	10 (8.9%)	3 (3.3%)	13 (6.4%)	
Other/unknown	10 (8.9%)	5 (5.4%)	15 (7.4%)	
Marital status (*N* = 6)				
Married	19 (17.0%)	18 (19.6%)	37 (18.1%)	0.202
Separated	6 (5.4%)	8 (8.7%)	14 (6.9%)	
Divorced	29 (25.9%)	18 (19.6%)	47 (23.0%)	
Widowed	16 (14.3%)	13 (14.1%)	29 (14.2%)	
Never married	36 (32.1%)	22 (23.9%)	58 (28.4%)	
Other	6 (5.4%)	13 (14.1%)	19 (9.3%)	
Lives alone				
No	67 (59.8%)	63 (68.5%)	130 (63.7%)	0.207
Yes	45 (40.2%)	28 (30.4%)	73 (35.8%)	
Unknown/missing	0 (0.0%)	1 (1.1%)	1 (0.5%)	
Household size, mean (standard deviation)	2.295 (1.493)	2.578 (1.748)	2.421 (1.613)	0.216
Hypertension reported	75 (67.0%)	56 (60.9%)	131 (64.2%)	0.366
Diabetes reported	59 (52.7%)	42 (45.7%)	101 (49.5%)	0.318
ADI National Rank				
76–100 (most disadvantaged)	102 (91.1%)	84 (91.3%)	186 (91.2%)	0.635
51 to 75	0 (0.0%)	0 (0.0%)	0 (0.0%)	
26 to 50	4 (3.6%)	5 (5.4%)	9 (4.4%)	
25 (least disadvantaged)	4 (3.6%)	1 (1.1%)	5 (2.5%)	
Suppressed/missing	2 (1.8%)	2 (2.2%)	4 (2.0%)	

Program retention and engagement among intervention participants were high. Of the 125 enrolled participants, 112 (89%) completed the 6-month assessment and 105 (84%) completed the 12-month assessment (see Fig. 1). All intervention participants completed at least one RD-led coaching session, and a majority (82%) completed all three planned sessions. Attendance at group-based educational sessions was lower, with 41% attending at least one session. Overall satisfaction with the intervention was high, with 97% of participants rating their experience as positive or very positive.^22^

Results are presented first for prespecified between-group comparisons at 6 months, followed by within-intervention cohort analyses and exploratory subsample analyses.

### Between-Group Outcomes: Intervention vs. SNAP-Only Comparison (Baseline to 6 Months)

#### Primary Outcomes

Between-group analyses comparing changes from baseline to 6 months showed differential effects across outcomes (see Table 3). Relative to the SNAP-only comparison cohort, intervention participants experienced significantly greater improvements in FV intake. Mean intake increased from 2.3 to 2.6 cups/day in the intervention cohort, compared to a negligible increase in the comparison group, resulting in a statistically significant between-group difference in change of + 0.3 cups/day (95% CI, 0.1 to 0.5; *p* = 0.002).

**Table 3:** Change in Outcomes Associated with the Intervention

Outcomes	Intervention cohort (*N* = 112)			Comparison cohort (*N* = 92)			Change in outcome associated with intervention	*p*-value^2^
	Baseline	6-month	Change^1^	Baseline	6-month	Change^1^		
Low or very low food security, %	58.9%	46.4%	−12.5%	52.2%	47.8%	−4.4%	−8.2% [−22.4%, 6.1%]	0.260
Fruit and vegetable intake in cup equivalents per day								
With fries	2.4	2.7	0.3	2.4	2.5	0.1	0.3 [0.1,0.5]	0.002
Without fries	2.3	2.6	0.3	2.3	2.3	0.0	0.3 [0.1, 0.5]	0.002
Total loneliness score (UCLA 3-Item Scale; 3–9)	4.8	4.6	−0.3	4.5	4.8	0.3	−0.6 [−1.1,−0.1]	0.022
Moderate or severe depression, %	29.5%	14.3%	−15.2%	23.9%	19.6%	−4.3%	−10.8%[−22.5%, 0.9%]	0.070

(1) To model the change in outcome associated with the intervention, we developed a panel analytic dataset with two observations for each person, one for the baseline and another for the 6-month periods. We defined indicators of the two periods (post = 1 if 6-month and post = 0 if baseline) and intervention/comparison indicator (intervention = 1 or 0)

(2) To estimate the change in outcome associated with the intervention, we estimated a linear regression (with individual fixed effects) of the outcome on the interaction of intervention and post indicators. The coefficient of the interaction term is the estimate of the change in outcome associated with the intervention

(3) Low or very low food security is determined through utilization of the U.S. Household Food Security Survey Module: Six-Item Short Form

Economic Research Service, USDA

(4) Fruit and vegetable intake is measured in cup equivalents per day using the Dietary Screener Questionnaire (DSQ) Fruit and Vegetable Guide

(5) Total loneliness score is measured on a scale of 3–9 using the UCLA 3-Item Loneliness scale survey instrument where 3 indicates “Least lonely” and 9 indicates “Most lonely”

(6) Moderate or severe depression is defined as a Patient Health Questionnaire-9 (*PHQ-9*) score of 11 or greater

(7) The total *N* for each cohort includes individuals that were alive and enrolled for the entire duration of their study follow-up periods with a combine cohort total of *N* = 204. The no. of individuals in each calculation in the above table varies by outcome. This is due to missingness from respondent non-answer. The total numbers noted for each outcome, respectively, are as follows: *N* = 204, *N* = 201, *N* = 201, *N* = 204, and *N* = 204

Loneliness also improved more in the intervention cohort than in the comparison group over the same interval. Mean loneliness scores decreased from 4.8 to 4.6 among intervention participants while increasing from 4.5 to 4.8 among comparison participants, corresponding to a significant between-group difference in change of −0.6 points (95% CI, −1.1 to −0.1; *p* = 0.022).

Between-group differences for FI and depressive symptoms favored the intervention but did not reach statistical significance. The prevalence of low or very low food security declined by 12.5 percentage points in the intervention cohort compared with a 4.4-percentage-point decline in the comparison cohort (between-group difference −8.2 percentage points; *p* = 0.260). Moderate-to-severe depressive symptoms declined by 16.2 percentage points in the intervention cohort versus 4.3 percentage points in the comparison cohort (adjusted between-group difference of −10.8 percentage points;* p* = 0.070).

#### Healthcare Utilization and Costs

Between-group analyses of healthcare utilization and costs were conducted over 6-, 12-, and 18-month periods (see Table 4). At 6 months, changes in utilization were small and did not differ between groups. Primary care visits decreased slightly in the intervention cohort (–0.09 visits) and increased slightly in the comparison cohort (+ 0.02), yielding a non-significant adjusted difference of –0.11 visits (*p* = 0.517). Emergency department visits declined marginally in the intervention cohort (–0.04) and remained stable in the comparison group, and hospitalizations changed minimally in both groups. Cost changes for primary care (–$5.02), emergency department visits (–$28.35), and hospitalizations (+ $1026.60) were not statistically significant. Utilization patterns remained relatively stable at 12 and 18 months, and no statistically significant between-group changes in utilization or costs were observed at any time point.

**Table 4:** Between Cohort Changes in Healthcare Utilization

Outcomes	Intervention cohort (*N* = 125)			Comparison cohort (*N* = 115)			Change in outcome associated with intervention	*p*-value^2^
	6 months before	6 months after	Change^1^	6 months before	6 months after	Change^1^		
**Primary care visits**								
** Number of visits**	1.27	1.18	−0.09	0.81	0.83	0.02	−0.11 [−0.43, 0.21]	0.517
** Costs of visits ($)**	140.62	137.42	−3.20	92.32	94.14	1.82	−5.02 [−41.03, 30.98]	0.784
**ED Visits**	0.54	0.50	−0.04	0.32	0.33	0.01	−0.04 [−0.30, 0.21]	0.753
** Costs ($)**	439.18	435.11	−4.07	269.79	294.07	24.28	−28.35 [−261.85, 205.14]	0.811
**Hospitalizations**	0.12	0.11	−0.01	0.23	0.14	−0.09	0.09 [−0.07, 0.25]	0.281
** Costs ($)**	1763.83	1688.26	−75.57	3553.93	2451.77	−1102.16	1026.60 [−1735.96, 3789.16]	0.465
	12 months before	12 months after	Change^1^	12 months before	12 months after	Change^1^		
**Primary care visits**	2.26	2.23	−0.03	1.49	1.6	0.11	Primary Care Visits	0.526
** Costs ($)**	250.32	257.1	6.78	170.82	184.71	13.89	Costs ($)	0.780
**ED visits**	0.98	0.84	−0.14	0.63	0.72	0.09	ED Visits	0.180
** Costs ($)**	819.25	733.8	−85.45	499.51	655.16	155.65	Costs ($)	0.126
**Hospitalizations**	0.19	0.26	0.07	0.3	0.32	0.02	0.05 [−0.17, 0.26]	0.672
** Costs ($)**	3268.86	3552.47	283.61	4699.37	5248.26	548.89	−265.29 [−3881.77, 3351.20]	0.885
	12 months before	18 months after^†^	Change^1^	12 months before	18 months after^†^	Change^1^		
**Primary care visits**	2.26	2.24	−0.02	1.49	1.38	−0.11	0.09 [−0.44, 0.62]	0.736
** Costs ($)**	250.32	274.42	24.10	170.82	164.15	−6.67	30.78 [−39.73, 101.28]	0.391
**ED visits**	0.98	0.82	−0.16	0.63	0.7	0.07	−0.24 [−0.61, 0.12]	0.193
** Costs ($)**	819.25	694.2	−125.05	499.51	614.59	115.08	−240.12 [−559.01, 78.76]	0.139
**Hospitalizations**	0.19	0.26	0.07	0.3	0.29	−0.01	0.08 [−0.10, 0.26]	0.400
** Costs ($)**	3268.86	3567.91	299.05	4699.37	6847.07	2147.70	−1848.66 [−7938.08, 4240.76]	0.550

#### Within Intervention Cohort Outcomes (Baseline to 6 and 12 Months)

Within-group analyses were conducted to describe longitudinal changes among intervention participants who completed the 12-month follow-up assessments (see Table 5). Among participants who completed the 12-month follow-up (*N* = 105), the prevalence of low or very low food security declined from 58.1% at baseline to 43.8% at 6 months and 41% at 12 months (adjusted change –14.3 percentage points at 6 months, *p* = 0.009; –17.1 percentage points at 12 months, *p* = 0.003). FV intake increased from 2.3 cups/day at baseline to 2.6 at 6 months and 2.7 at 12 months (adjusted change + 0.3 cups/day at 6 months and 0.4 cups/day at 12 months, both *p* < 0.001).

**Table 5:** Change in Outcomes Associated with Follow-up period for the Intervention Group

Outcomes (*N* = 105)	Baseline	Month 6	Month 12	Adjusted change from months 1–6 [CI] (*p*-value)	Adjusted change from months 1–12 [CI] (*p*-value)
Low or very low food security, %	58.1%	43.8%	41.0%	−14.3% [−24.9%, −3.7%] (0.009)	−17.1% [−28.4%, −5.9%] (0.003)
Fruit and vegetable intake in cup equivalents per day					
With fries	2.4	2.7	2.8	0.3 [0.2, 0.5] (< 0.001)	0.4 [0.2, 0.6] (< 0.001)
Without fries	2.3	2.6	2.7	0.3 [0.2, 0.5] (< 0.001)	0.4 [0.2, 0.6] (< 0.001)
Total loneliness score (UCLA 3-Item Scale; 3–9)	4.8	4.4	4.3	−0.4 [−0.8, 0.0] (0.057)	−0.4 [−0.8, −0.1] (0.015)
Moderate or severe depression, %	28.6%	12.4%	14.3%	−16.2% [−24.3%, −8.1%] (< 0.001)	−14.3% [−22.5%, −6.0%] (0.001)
Fatigue	20.8	24.3	23.9	3.4 [1.4, 5.5] (0.001)	3.1 [0.5, 5.7] (0.019)
Weight in kg	96.0	94.7	94.0	−1.3 [−2.2, −0.4] (0.006)	−2.0 [−3.5, −0.4] (0.015)
Body mass index (BMI)	35.8	35.6	35.4	−0.2 [−0.7, 0.2] (0.322)	−0.4 [−1.1, 0.2] (0.207)
Waist circumference in cm	111.9	110.6	109.4	−1.3 [−3.2, 0.6] (0.167)	−2.6 [−4.6, −0.5] (0.015)
Systolic blood pressure	132.0	128.5	129.1	−3.6 [−7.7, 0.6] (0.090)	−2.9 [−7.6, 1.8] (0.225)
Diastolic blood pressure	83.4	79.0	78.3	−4.5 [−6.9, −2.0] (0.001)	−5.1 [−8.0, −2.3] (0.001)

(1) To model the change in outcome associated with the follow-up periods for the intervention group only, we developed a panel analytic dataset with three observations for each person, one for the baseline, one for the 6-month follow-up, and another for the 12-month follow-up period. We defined 3 indicators of follow-up groups (post1 = 1 if 6-month and post = 0 if baseline; post2 = 1 if 12-month and post = 0 if 6-month; post3 = 1 if 12-month and post = 0 if baseline)

(2) To estimate the change in outcome associated with the follow-up periods, we estimated separate linear regressions with individual fixed effects on indicators of the follow-up period. The coefficient of the follow-up period indicator is the estimate of the change in outcome associated with the follow-up period

(3) Please see Table * notes 3–6 for explanation of outcome values of food security to depression

(4) The Functional Assessment of Chronic Illness Therapy – Fatigue Scale (FACIT-Fatigue) is a 13-item measure that assesses self-reported fatigue and its impact upon daily activities and function. This survey tool was used to calculate fatigue score; a higher score is associated with better quality of life (QOL)

(5) From weight to blood pressure, outcomes in column 1 were assessed during in-person consultations used standardized protocols

(6) The total *N* for each period includes the intervention cohort of individuals that were alive and enrolled for the entire duration of their study follow-up periods from baseline to 12-month follow-up (*N* = 105). The no. of individuals in each calculation in the above table varies by outcome. This is due to missingness from respondent non-answer. The total numbers noted for each outcome, respectively are as follows: *N* = 105, *N* = 97, *N* = 97, *N* = 104, *N* = 105, *N* = 105, *N* = 90, *N* = 88, *N* = 89, *N* = 91, and *N* = 91

Psychosocial outcomes improved within the intervention cohort over 12 months. Moderate-to-severe depressive symptoms declined from 28.6% to 12.4% at 6 months and 14.3% at 12 months (adjusted change –16.2 percentage points at 6 months; –14.3 percentage points at 12 months, both *p* = 0.001). Mean loneliness scores declined from 4.8 at baseline to 4.4 at 6 months and 4.3 at 12 months, with the adjusted change approaching significance at 6 months (–0.4 points; *p* = 0.057) and reaching statistical significance at 12 months (–0.5 points; *p* = 0.001).

#### Secondary Outcomes and Exploratory Subsample Analyses (Intervention Cohort)

Secondary outcomes were assessed for the intervention cohort at baseline, 6 months, and 12 months (see Table 5). FACIT-F scores improved from 20.8 at baseline to 24.3 at 6 months and 23.9 at 12 months (adjusted change + 3.4 points at 6 months; + 3.1 points at 12 months, *p* = 0.019), exceeding the 3-point threshold for clinical significance.^44^ Weight decreased by 1.3 kg at 6 months (*p* = 0.006) and 2.0 kg at 12 months (*p* = 0.015). Waist circumference declined by 1.3 cm at 6 months (*p* = 0.167) and 2.6 cm at 12 months (*p* = 0.015).

Exploratory analyses examined BP changes within the intervention cohort, including a subsample of participants with elevated baseline BP (*n*=73) (see Table 6). In the intervention cohort, DBP decreased by 4.5 mmHg at 6 months and 5.1 mmHg at 12 months (both *p*=0.001), while SBP reductions were smaller and not statistically significant. Among participants with elevated baseline BP (*n*=73), SBP decreased by 8.6 mmHg (*p*=0.003) and DBP decreased by 8.5 mmHg (*p*<0.001).

**Table 6:** Change in Outcomes Associated with Follow-up Period for the Intervention Group Subsample of Individuals with Systolic > 130 or Diastolic > 80 at Baseline

Outcomes (*N *= 73)	Baseline	Month 6	Month 12	Adjusted change months 1–6 [CI] (*p*-value)	Adjusted change months 7–12 [CI] (*p*-value)	Adjusted change months 1–12 [CI] (*p*-value)
Moderate or severe depression, %	31.5%	13.7%	16.4%	−17.8% [−26.8%, −8.8%] (< 0.001)	2.7% [−4.0%, 9.5%] (0.420)	−15.1% [−24.4%, −5.8%] (0.002)
Low or very low food security, %	60.3%	45.2%	42.5%	−15.1% [−28.4%, −1.7%] (0.027)	−2.7% [−15.1%, 9.6%] (0.659)	−17.8% [−32.1%, −3.6%] (0.015)
Fruit and vegetable intake in cup equivalents per day						
With fries	2.5	2.8	2.8	0.3 [0.1, 0.5] (0.008)	0.0 [−0.1, 0.2] (0.599)	0.4 [0.1, 0.6] (0.007)
Without fries	2.4	2.7	2.7	0.3 [0.1, 0.6] (0.007)	0.0 [−0.1, 0.2] (0.627)	0.4 [0.1, 0.6] (0.006)
Total loneliness score (UCLA 3-Item Scale; 3–9)	4.8	4.4	4.6	−0.4 [−0.9, 0.0] (0.071)	0.2 [−0.3, 0.6] (0.472)	−0.3 [−0.7, 0.2] (0.215)
Weight in kg	97.1	96.3	95.0	−0.8 [−1.9, 0.2] (0.123)	−1.2 [−3.1, 0.7] (0.205)	−2.0 [−4.1, 0.0] (0.055)
Body mass index (BMI)	36.3	36.2	35.8	−0.1 [−0.7, 0.5] (0.712)	−0.3 [−1.0, 0.4] (0.337)	−0.5 [−1.4, 0.5] (0.337)
Waist circumference in cm	112.8	112.4	111.4	−0.4 [−2.8, 2.0] (0.744)	−1.0 [−2.5, 0.5] (0.187)	−1.4 [−4.1, 1.3] (0.307)
Systolic blood pressure	141.7	133.2	133.1	−8.5 [−12.8, −4.2] (< 0.001)	−0.1 [−6.8, 6.7] (0.984)	−8.6 [−14.0, −3.1] (0.003)
Diastolic blood pressure	88.6	82.5	80.1	−6.1 [−8.9, −3.3] (< 0.001)	−2.4 [−5.2, 0.5] (0.100)	−8.5 [−11.8, −5.2] (< 0.001)
Fatigue	20.5	24.1	22.7	3.6 [1.3, 5.8] (0.002)	−1.4 [−4.0, 1.1] (0.273)	2.1 [−1.0, 5.2] (0.176)

## DISCUSSION

### Principal Findings

In this non-randomized, matched cohort study, participation in a 12-month PRx program was associated with greater improvements in FV intake and loneliness compared with a SNAP-only comparison group at 6 months. Between-group differences for FI and depressive symptoms favored the intervention but did not reach statistical significance, likely reflecting limited power to detect changes in binary outcomes. In contrast, within-intervention cohort analyses demonstrated improvements in dietary, psychosocial, and selected clinical outcomes over 12 months. These within-group findings should be interpreted as descriptive and hypothesis-generating rather than causal.

The observed improvement in FV intake aligns with prior evaluations demonstrating dietary benefits of PRx programs among low-income adults with diet-sensitive chronic conditions.^46–50^ Notably, improvements in loneliness highlight a psychosocial benefit that is less frequently examined in PRx evaluations but may be relevant for populations experiencing FI and chronic disease.^19,49^ While reductions in FI and depressive symptoms were not statistically significant in between-group analyses, the direction and magnitude of effects were consistent with prior observational studies and suggest potential benefit that warrants further investigation.^49,50^

Although this study was not powered to formally test mechanisms of action, findings suggest that PRx may influence health through both nutritional and psychosocial pathways.^33,49^ The observed SBP and DBP reductions parallel those in smaller scale, higher-intensity clinical trials, suggesting that PRx can achieve clinically meaningful cardiometabolic change in community settings when fidelity is maintained.^51^ Improvements in loneliness, depressive symptoms, and fatigue may enhance motivation, self-management capacity, and engagement with dietary recommendations, complementing gains in food access and diet quality.^52–55^ These pathways are consistent with behavioral theory and prior qualitative work demonstrating increased social connection, perceived support, and confidence among participants in FIM interventions.^33,52–55^ Our 12-month follow-up period and matched comparison group distinguish this evaluation from many PRx studies that assess shorter-term change or lack control groups. Integration of produce delivery, individualized coaching, and community-based educational programming may have contributed to high engagement by addressing both access and motivation barriers, elements less common in voucher or delivery-only programs.^23,25^

Short-term healthcare utilization and costs were unchanged, aligning with prior literature suggesting that cost savings may require longer follow-up.^21,29^ These findings should be interpreted in the context of concurrent SNAP participation and broader economic conditions that may influence healthcare use in both cohorts.^56^

### Policy and Practice Implications

Theory informed PRx programs have the potential to address multilevel barriers to dietary change by combining food access with behavioral and psychosocial support.^32–34^ In this study, flexible delivery, individualized coaching, and community-located education appeared to enhance feasibility and participant engagement.^32^ Although short-term utilization effects were neutral, observed improvements in diet quality and psychosocial outcomes suggest potential long-term value, particularly if programs are embedded in sustainable reimbursement and delivery models.^18,48^ Including psychosocial outcomes as core PRx endpoints may better capture program impact, as improvements in mood, loneliness, and fatigue are closely linked to chronic disease self-management and engagement.^53,54^

### Strengths and Limitations

Strengths include a prospective design, matched comparison group, and theory-informed intervention, and high participant retention over 12 months. Qualitative and implementation data suggest that home delivery, flexible coaching modalities, accessible group education, and consistent contact with trusted RD facilitated retention by reducing logistical burden and improving perceived relevance. Limitations include the non-randomized design, limited power for some between-group comparisons, reliance on self-reported measures, and implementation within a single health system, which may limit generalizability. Within-group and subgroup analyses were exploratory and should not be interpreted as causal.

### Future Directions

This evaluation highlights several priority areas for future research. First, studies should further examine psychosocial mechanisms of action as drivers, including whether improvements in loneliness, depressive symptoms, and fatigue mediate changes in diet quality and chronic disease self-management. Second, comparative effectiveness studies are needed to identify the most impactful combinations of delivery mode, produce dose, and behavioral support, particularly in Medicaid populations. Third, a longer follow-up is required to test the durability of effects and potential downstream impacts on healthcare utilization and costs. Finally, implementation research should focus on scalability, sustainability, and financial strategies to support the integration of PRx into routine care while allowing for local adaptation.

## CONCLUSION

Participation in a 12-month PRx program was associated with improvements in diet quality and psychosocial outcomes among Medicaid-insured adults with chronic conditions. While causal inference is limited, these findings support PRx programs as a promising FIM approach that addresses both nutritional and psychosocial needs. Rigorous comparative studies with longer follow-up are needed to determine durability, mechanisms of action, and implications for sustainable implementation.

## Data Availability

The datasets during and/or analyzed during the current study available from the corresponding author on reasonable request.
